# Interceptive Orthodontics and Temporomandibular Joint Adaptations: Such Evidences?

**DOI:** 10.1155/2017/8953572

**Published:** 2017-04-26

**Authors:** Simona Tecco, Alberto Baldini, Enita Nakaš, Jasmina Primozic

**Affiliations:** ^1^Dental School, University Vita-Salute San Raffaele, Milan, Italy; ^2^University Tor Vergata, Rome, Italy; ^3^School of Dental Medicine, University of Sarajevo, Sarajevo, Bosnia and Herzegovina; ^4^University Clinical Centre of Ljubljana, Ljubljana, Slovenia

The research of the anatomy of the temporomandibular joint (TMJ) and its development during the growing period is far beyond the classical approach. TMJ area has been thought to be a reactive growth site, which makes it more interesting for research. During the period of mixed dentition, TMJ area is affected by a considerable amount of growth and adaptation, which can alter the jaws relationships. Also, a variable adaptation can occur during an interceptive functional orthodontic treatment. Nowadays, TMJ studies comprise various fields of medicine, starting from clinical investigations, up to the in vitro models, which are useful in studies of the anatomy and disorders of the two jaws. This is the reason why we have gathered researches that are closely related to TMJ. The* leitmotif* of the special issue is the anatomy of the jaws and their adaptions during the growth and development of individuals and the interceptive orthodontic treatments.

G. Perinetti and L. Contardo, on the basis of a literature review, explain the current evidence and controversies on the efficiency of interceptive orthodontics and conclude that more favorable response is seen when subjects are treated during their pubertal growth spurt, mostly in skeletal Class II patients (even though high individual responsiveness remains). They also clarify that no growth indicator may be considered to have a full diagnostic reliability to assess the pubertal growth spurt of a patient. Nevertheless, their use may still be recommended for increasing efficiency of interceptive orthodontics, in particular for skeletal Class II malocclusion.

Another paper by G. Perinetti et al. is more specifically dealing with a growth indicator of the timing of circumpubertal skeletal maturation (circumpubertal cervical vertebral maturation, CVM) to assess its relationship with the sagittal and vertical mandibular development. This is a cross-sectional study aimed at evaluating whether sagittal and vertical craniofacial growth has an association with the timing of circumpubertal skeletal maturation. A total of 320 subjects (160 females and 160 males) are included in the study (mean age, 12.3 ± 1.7 years; range, 7.6–16.7 years). These subjects were equally distributed in the circumpubertal cervical vertebral maturation (CVM) stages 2 to 5. Significant associations were seen only for Stage 3, where the mandibular to cranial base angle (i.e., the mandibular divergence with cranial base) results negatively when associated with age (*β* coefficient, −0.7), suggesting that the mandibular divergence (linked to TMJ development) may have an anticipated and delayed attainment of the pubertal CVM Stage 3. The clinical conclusion is that this association remains of a small entity, and it becomes clinically relevant only in extreme cases.

M. C. Sobral de Aguiar et al. present a review of studies performed to evaluate whether the gingival crevicular fluid (GCF) biomarkers in growing subjects reflect both the stages of individual skeletal maturation and the local tissue remodeling triggered by orthodontic force. However the conclusion is that, in spite of several investigations, the clinical applicability of the GCF method is still limited to further data needed to reach a full diagnostic utility of specific GCF biomarkers in interceptive orthodontics.

T. Lauc et al. investigated whether skeletal pattern of the growth can influence dental development and found that males with Class III skeletal pattern have faster dental development. Research results suggest that diversity of the skeletal pattern could be connected with the different timing of dental development.

J. Badrov et al. have found that changes in the development of permanent teeth during growth can occur in children with the congenitally missing permanent teeth (CMPT). They also reported that the dental age is significantly delayed in CMPT children compared to the nonaffected group; the mean differences are −0.57 ±  1.20 years and −0.61  ±  1.23 years in males and females, without difference between sexes.

A. H. Al-Ani et al. also declared that the tooth agenesis, especially in its severe forms, is often associated with various anomalies in other teeth, such as delays in development, ectopic eruption, reduction in tooth dimensions and morphology, shortened roots, taurodontism, and enamel hypoplasia. Also, the authors explained that the hypodontia patients tend to show with lower mandibular plane angles, associated with a smaller lower anterior face height and lip protrusion. Other features associated with hypodontia include shorter maxillary and mandibular lengths and a Class III skeletal relationship tendency.

In reference to interceptive functional orthodontic treatment, the following two papers are dealing with the efficacy of functional appliances in growing subjects.

A study of the comparison between the Activator-Headgear (AH) and the Twin Block (TB) treatments approaches in Class II division 1 malocclusion has been conducted by S. Spalj et al. Their results suggest that both AH and TB appliances contribute successfully to the correction of Class II division 1 malocclusion when compared to the untreated subjects with primarily dentoalveolar changes. The authors explained that the correction of malocclusion is made by retroclination of maxillary incisors and proclination of mandibular incisors, the latter being significantly more evident in the TB group, and with the increase of effective mandibular length that was also more evident in the TB group.

Another clinical study to appraise the factors affecting the wearing time and patient's behavior during a functional treatment with a newly designed reverse pull headgear is presented by N. Ozkalayci and O. Cicek. They found that patients wore the new reverse pull headgears mostly during the night, due to problems related to aesthetic appearance, and during the weekends.

Two interesting papers are dealing with the study of the morphology of TMJ on 3D-imaging.

Cone-Beam Computerized Tomography (CBCT) represents widely used diagnostic image system, and it is based on 3D visualization. The study by S. Caruso et al. analyzed the recent literature about TMJ visualization on CBCT imaging. Sources included PubMed from June 2008 to June 2016. Eleven articles were finally included in the qualitative synthesis. The main topics treated in the studies are the volume and surface of the mandibular condyle, the bone changes on the cortical surface, the morphological asymmetry between the two condyles, and the optimum position of the condyle in the glenoid fossa. In particular, the conclusion of this review is that CBCT 3D imaging allows the calculation of volume and surface of the mandibular condyle, the calculation of its linear dimensions (height and length), and the measurement of the intra-articular space to clarify the position of the condyle in the glenoid fossa.

S. Mummolo et al. presented data about the 3D Tele Motion Tracking (3D-TMT), as a useful tool for facial analysis. A group of 40 patients (20 males and 20 females; mean age, 12–18 years) was included in the study. The measurements obtained by the 3D-TMT and by a traditional 2D radiological analysis were compared for each subject. The 3D-TMT system values resulted slightly higher, statistically significant, than the values obtained on radiographs; nevertheless, their correlation resulted very high, and the Dahlberg errors resulted in being always lower than the mean difference between the 2D and 3D measurements. The authors suggest that a clinician should always use, during the clinical monitoring of a patient, the same method (2D or 3D), to avoid comparing different millimeter magnitudes in the dimensions of a face.

Overall, studies show that the maxillary and mandibular area are affected by significant morphological changes during the period of the circumpubertal spurt and, therefore, can undergo a significant remodeling during that time, also in response to orthodontic interceptive devices. Although there are several methods in the literature useful to predict the coming of the circumpubertal spurt, none of them is infallible, and future studies are needed to clarify this point. Also, the dental age is influenced by several variables, such as the agenesis of the permanent elements. The clinical monitoring of facial changes during the interceptive therapy seems possible using 3D imaging techniques, such as CBCT images, and also through noninvasive methods for the study of facial structures (as, e.g., the 3D Tele Motion Tracking).

All presented papers share the same interest related to the anatomy of stomatognathic system during the growth development.

All of the above presented papers along with the latest technologies have contributed to advancing knowledge of anatomical structures and their changes during growth and interceptive orthodontics treatment. Their role in the pathogenesis of diseases and disorders of different origin is going to be, even partially, clarified.

*Simona Tecco* *Alberto Baldini* *Enita Nakaš* *Jasmina Primozic*

